# Discriminatory ability of visceral adiposity index as an indicator for modeling cardio-metabolic risk factors in pediatric population: the CASPIAN-V study

**DOI:** 10.15171/jcvtr.2019.46

**Published:** 2019-10-24

**Authors:** Hanieh-Sadat Ejtahed, Roya Kelishadi, Shirin Hasani-Ranjbar, Pooneh Angoorani, Mohammad Esmaeil Motlagh, Gita Shafiee, Hasan Ziaodini, Majzoubeh Taheri, Mostafa Qorbani, Ramin Heshmat

**Affiliations:** ^1^Obesity and Eating Habits Research Center, Endocrinology and Metabolism Clinical Sciences Institute, Tehran University of Medical Sciences, Tehran, Iran; ^2^Department of Pediatrics, Child Growth and Development Research Center, Research Institute for Primordial Prevention of Non-communicable Disease, Isfahan University of Medical Sciences, Isfahan, Iran; ^3^Department of Pediatrics, Ahvaz Jundishapur University of Medical Sciences, Ahvaz, Iran; ^4^Chronic Diseases Research Center, Endocrinology and Metabolism Population Sciences Institute, Tehran University of Medical Sciences, Tehran, Iran; ^5^Health Psychology Research Center, Education Ministry, Tehran, Iran; ^6^Office of Adolescents and School Health, Ministry of Health and Medical Education, Tehran, Iran; ^7^Non-communicable Diseases Research Center, Alborz University of Medical Sciences, Karaj, Iran; ^8^Endocrinology and Metabolism Research Center, Endocrinology and Metabolism Clinical Sciences Institute, Tehran University of Medical Sciences, Tehran, Iran

**Keywords:** Adolescents, Children, Metabolic Syndrome, Visceral Adiposity Index

## Abstract

***Introduction:*** The purpose of this study was to obtain the cutoff points of visceral adiposity index (VAI), a new marker of indirect evaluation of visceral fat, to assess its association with metabolic syndrome (MetS) in a population of children and adolescents.

***Methods:*** This cross sectional study was conducted on children and adolescents aged 7-18 years attended in the fifth phase of a national school-based surveillance survey. The odds ratio (OR) of cardiometabolic risk factors across tertile categories of VAI was determined using the logistic regression models and the valid cut-off values of VAI for predicting MetS was obtained using the receiver operation characteristic (ROC) curve analysis.

***Results:*** A total of 3843 students (52.3% boys, 12.3 [12.2-12.4] years) were included in the analysis. The mean of VAI was significantly higher in participants who had MetS (2.60 [2.42-2.78] vs 1.22 [1.19-1.25]; *P* <0.001). Participants in the third tertile compared to the first tertile category of VAI had higher odds of abdominal obesity (OR: 1.77, 95% CI: 1.43-2.20), impaired fasting blood glucose (OR: 2.00, 95% CI: 1.28-3.13) and low high-density lipoprotein cholesterol (OR: 15.93, 95% CI: 12.27-20.66). The cut-off points of the VAI for predicting MetS were 1.58, 1.30 and 1.78 in total population, boys and girls, respectively.

***Conclusion:*** We determined the cut-off points of VAI as an easy tool for detecting MetS in children and adolescents and demonstrated that VAI is strongly associated with MetS. Prospective longitudinal studies are suggested to show the possible efficiency of the VAI as a predictor of MetS in pediatrics.

## Introduction

Worldwide growing of metabolic syndrome (MetS) as a warning of chronic diseases in children and adolescents will cause great suffering and overwhelm medical treatment systems. Since MetS in childhood increases the risk of cardiovascular diseases in adulthood, diagnosis and treatment of at risk children could be very valuable.^1-3^ Visceral obesity is associated with increased risk of metabolic abnormalities including insulin resistance, dyslipidemia, hypertension, atherosclerosis, diabetes and higher mortality rate.^4^

Although dual-energy X-ray absorptiometry, computed tomography and ultrasound are accurate methods for measurement of visceral adiposity and may use as a clinical practice,^5,6^ but using them as routine methods has some limitations. The high cost, radiation exposure, requirement to intensive training and low availability for most clinicians make these methods challenging to use in clinical settings especially in children and adolescents.^7^ Therefore, surrogate indices of visceral adiposity can be useful in this context. Visceral adiposity index (VAI) has been proposed as a new method for evaluation of fat distribution and function. This index is a gender-specific mathematical model, derived from simple anthropometric measurements (body mass index [BMI]and waist circumference [WC]) and functional parameters (triglycerides [TG] and high-density lipoprotein cholesterol [HDL-C]) and it may work better compared to single index.^8^ It has been shown that VAI is positively correlated with insulin resistance, functional glycemic disorders and increased risk of cardiovascular diseases.^9-11^ Changes in body fat during childhood are associated with development of MetS. However, the information about the value of VAI is particularly limited in children and adolescents and a valid cut-off value has not been determined in Iranian pediatrics. The aim of this study is to calculate VAI in a representative sample of Iranian children and adolescents and to obtain valid cut-off values for met­abolic disorders.

## Materials and Methods

### Study population

This cross sectional study was conducted on Iranian children and adolescents aged 7-18 years participated in the fifth phase of a school-based surveillance survey entitled “Childhood and Adolescence Surveillance and Prevention of Adult Non-communicable Disease” (CASPIAN) in 2015. The detailed descriptions of the survey have been reported previously.^12^ Briefly, sample selection was done by multistage, stratified cluster sampling method from both urban and rural areas of 30 provinces in Iran. Sampling within each province was done according to the proportional to size method regarding the place of residence (urban or rural) and level of education (primary and secondary) and with equal number of boys and girls. The desired sample size in each province (about 480 students) was achieved using cluster sampling with equal cluster sizes. Totally, 14,138 students were assessed at national level that resulted in a good estimate of all selected risk factors. In each province, 14 clusters out of 48 clusters (finally 3843 students) were randomly selected for biochemical experiments. Fasting blood samples were collected from 3843 participants.^13^

### Clinical and biochemical measurements

Weight was measured using a scale placed on a flat ground with the precision of 0.1 kg while subjects minimally clothed without shoes. Height was measured to the nearest 0.5 cm without shoes using a tape meter. Waist circumference was measured to the nearest 0.1 cm, using non-stretchable tape meter.^14,15^ Body mass index was calculated as weight in kg divided by the square of height in m^2^. World health organization (WHO) growth curves was used for categorizing of BMI; overweight was defined as age and gender-specific BMI of 85^th^ to 95^th^ percentiles, and obesity as age and gender-specific BMI of >95^th^ percentiles. Abdominal obesity was defined as WC above 90^th^ percentile.^16^

Fasting blood samples were taken after 12 to 14 hours of overnight fast. Fasting blood glucose (FBS), total cholesterol (TC), low-density lipoprotein cholesterol (LDL-C), HDL-C, and TG were measured using enzymatic method by Hitachi auto-analyzer (Tokyo, Japan). Blood pressure (BP) was measured twice on the right arm, in the sitting position after 15 minutes of rest using a standardized mercury sphygmomanometer. The first and fifth Korotkoff sounds were considered as systolic blood pressure (SBP) and diastolic blood pressure (DBP), respectively. The mean of the two measurements was recorded as BP.^17^

The assessment of living area, socioeconomic status, physical activity, and screen time has been described previously.^18^

### Definition of terms

In this study VAI was calculated as follows^19^:

$$\begin{array}{l} Males:VAI=(\frac{WC}{39.68+(1.88\times BMI)})\times (\frac{TG}{1.03})\times (\frac{1.31}{HDL})\\ Females:VAI=(\frac{WC}{39.58+(1.89\times BMI)})\times (\frac{TG}{0.81})\times (\frac{1.52}{HDL}) \end{array}$$

In the formula, WC was introduced in cm, BMI in kg/m^2^, TG and HDL in mmol/L.

Metabolic syndrome was defined based on the modified version of Adult Treatment Panel III (ATP III) criteria for the pediatric age group. Individuals who had at least three of the following criteria were classified as having MetS: 1- TG concentration of 100 mg/dL or greater; 2- HDL-C concentration of 40 mg/dL or less (except in boys 15–19 y old which cut-off is < 45 mg/dL); 3- FBS concentration of 100 mg/dL or greater; 4- WC > 90th percentile (abdominal obesity); and 5- either SBP or DBP greater than the 90th percentile for age, gender, and height.^20^

### Statistical analysis

Data were presented as the mean (95% CI) or number (percentage). The normality of distribution was checked for all variables. The independent sample t-test was used to compare continuous variables and the chi-square test was used to compare proportions between boys and girls.

Associations between VAI and metabolic characteristics were tested by Pearson correlation. Mean of VAI in participants based on having MetS or not and having different number of MetS risk components was compared by Independent samples t test and one-way ANOVA.

The odds ratio (OR) of cardiometabolic risk factors including general and abdominal obesity, impaired fasting glucose, elevated BP, hypertriglyceridemia, low HDL-C, and high LDL-C across tertile categories of VAI was determined using the logistic regression models. Tertiles categories are provided for cross-classification of participants. In this classification, the first group was considered as a reference group to compare with other groups. Binary logistic regression models included a dichotomous outcome (for example, obesity; yes or no) and tertiles of VAI in 3 models; model 1: crude model, model 2: adjusted for age, gender, living area, socioeconomic status, physical activity, and screen time and model 3: additional adjustment for BMI. Data are presented as OR with 95% CI.

To estimate valid cut-off values of VAI for predicting MetS, the receiver operation characteristic (ROC) curve analysis was done with an estimation of the sensitivity and specificity. Analyses were performed separately for gender and age groups. The estimated optimal cut-points were determined using the minimum value of which represents the maximum sum of sensitivity and specificity. The area under curve (AUC) shows the ability of VAI cut-off points to discriminate pediatrics with and without metabolic syndrome accurately. Analyses were done using STATA version 11.0 (STATA Corp LP. Package, College Station, TX, USA). P-values less than 0.05 were considered as statistically significant.

## Results

In this study 3843 students (52.3% boys) with mean (95% CI) age of 12.3 (12.2-12.4) years were included in the analysis. According to the WHO criteria, 9.4% (8.7% of boys and 10.2% of girls) were overweight and 11.4% (12.5% of boys and 10.3% of girls) were obese. Abdominal obesity was observed in 21.1% of students (21.6% of boys and 20.5% of girls). The characteristics of study population according to gender are presented in Table 1. Briefly, boys presented higher values of age, weight, height, waist, BP (*P* < 0.001) and FBG (*P* < 0.05), but lower values of TC, LDL-C (*P* < 0.05) and VAI (*P* < 0.001). The bivariate correlation coefficients between VAI and metabolic characteristics are shown in Table 2. In these analyses, most variables including weight, height, BMI, WC, FBS, DBP, TG, TC and HDL-C were significantly associated with VAI in total participants (*P* < 0.01). Although, considering the clinical significance of these correlations, the correlations between weight, height, WC, FBS, DBP, TC and VAI were weak. BMI and HDL-C had moderate correlation with VAI (r = 0.38 and r = -0.48; *P* < 0.05, respectively) and perfect correlation was only observed between TG and VAI (r = 0.84, *P* < 0.01). Mean of VAI according to having MetS or not and having different numbers of MetS components, is illustrated in Table 3. The mean of VAI was significantly higher in participants (both boys and girls) who had MetS (*P* < 0.001).

**Table 1:** Characteristics of the study population: the CASPIAN-V study

**Variables**	**Total (n=3843)**	**Boys (n=2012)**	**Girls (n=1831)**	***P*** value*
Age (y)	12.3 (12.2-12.4)	12.4 (12.3-12.5)	12.2 (12.1-12.3)	<0.001
Weight (kg)	41.4 (40.8-41.9)	42.4 (41.6-43.2)	40.4 (39.7-41.1)	<0.001
Height (cm)	146.6 (146.0-147.2)	148.1 (147.3-148.9)	144.9 (144.2-145.6)	<0.001
BMI (kg/m^2^)	18.5 (18.3-18.6)	18.5 (18.3-18.7)	18.5 (18.3-18.7)	0.50
Waist (cm)	66.7 (66.3-67.1)	67.6 (67.0-68.2)	65.7 (65.2-66.2)	<0.001
SBP (mm Hg)	99.2 (98.8-99.6)	99.5 (98.9-100.1)	98.7 (98.1-99.3)	<0.001
DBP (mm Hg)	63.8 (63.5-64.1)	64.1 (63.6-64.6)	63.5 (63.0-64.0)	0.004
FBG (mg/dL)	91.6 (91.2-92.0)	92.1 (91.5-92.7)	91.2 (90.7-91.7)	0.027
TG (mg/dL)	88.0 (86.6-89.4)	87.1 (85.1-89.1)	89.0 (86.9-91.1)	0.20
TC (mg/dL)	153.8 (152.9-154.7)	152.9 (151.7-154.1)	154.8 (153.6-156.0)	0.03
HDL-C (mg/dL)	46.2 (45.9-46.5)	46.2 (45.7-46.6)	46.1 (45.6-46.5)	0.80
LDL-C (mg/dL)	90.3 (89.6-91.0)	89.3 (88.3-90.3)	90.8 (89.8-91.8)	0.03
VAI	1.29 (1.26-1.32)	1.03 (0.99-1.06)	1.58 (1.53-1.63)	<0.001

Data are expressed as mean (95% CI). * *P* values compared the characteristics of between boys and girls using Independent samples *t* test. BMI, body mass index; SBP, systolic blood pressure; DBP, diastolic blood pressure; FBG, fasting blood glucose; TC, total cholesterol; TG, triglycerides; LDL-C, low-density lipoprotein cholesterol; HDL-C, high-density lipoprotein cholesterol; VAI, visceral adiposity index.

**Table 2:** Bivariate correlation coefficients between visceral adiposity index and metabolic characteristics: the CASPIAN-V study

**Variables**	**Total**	**Boys**	**Girls**
Weight (kg)	0.07^**^	0.12**	0.08^**^
Height (cm)	0.08^**^	0.13**	0.10^**^
BMI (kg/m^2^)	0.38*	0.59*	0.33*
Waist (cm)	0.15^**^	0.20**	0.17^**^
SBP (mm Hg)	0.03	0.08**	0.02
DBP (mm Hg)	0.04^*^	0.07**	0.03
FBG (mg/dL)	0.12^**^	0.14**	0.15^**^
TG (mg/dL)	0.84^**^	0.87**	0.91^**^
TC (mg/dL)	0.11^**^	0.13**	0.09^**^
HDL-C (mg/dL)	-0.48^**^	-0.49**	-0.53^**^
LDL-C (mg/dL)	0.01	0.03	-0.02

*P < 0.05. **P < 0.01 using Pearson correlation test. BMI, body mass index; SBP, systolic blood pressure; DBP, diastolic blood pressure; FBG, fasting blood glucose; TC, total cholesterol; TG, triglycerides; LDL-C, low-density lipoprotein cholesterol; HDL-C, high-density lipoprotein cholesterol; VAI, visceral adiposity index.

**Table 3:** Mean of visceral adiposity index by Mets and numbers of MetS components: the CASPIAN-V study

	**Total**	**Boys**	**Girls**
MetS						
Yes	2.60 (2.42-2.78)	2.11 (1.87-2.35)	3.27 (2.95-3.59)
No	1.22 (1.19-1.25)	0.96 (0.93-0.99)	1.50 (1.46-1.54)
*P* value	<0.001	<0.001	<0.001
MetS components			
0	0.82 (0.80-0.84)	0.62 (0.60-0.63)	0.99 (0.97-1.01)
1	1.22 (1.18-1.25)	0.97 (0.94-1.00)	1.54 (1.48-1.60)
2	1.99 (1.91-2.07)	1.54 (1.46-1.62)	2.52 (2.38-2.65)
≥3	2.60 (2.42-2.78)	2.11 (1.87-2.35)	3.27 (2.95-3.59)
*P* value	<0.001	<0.001	<0.001

Data are expressed as mean (95% CI). * *P* values compared the visceral adiposity index among groups using Independent samples t test or ANOVA. Metabolic syndrome: ATP-III criteria; Abdominal obesity: WC> 90th percentile; Low HDL: HDL< 40 mg/dL (except in boys 15–19 y old, that cut-off was < 45 mg/dL); High TG: TG>100 mg/dL; High FBG: FBG > 100 mg/dL; High blood pressure: BP> 90th (adjusted by age, sex, height). MetS components: 0, no risk; 1, one risk; 2, two risks; ≥3, at least three risks.

Odds ratio and 95% confidence interval for cardiometabolic risk factors across tertile categories of VAI are provided in Table 4. Participants in the third tertile compared to the first tertile category of VAI, had higher risk of abdominal obesity (OR: 1.69, 95% CI: 1.39–2.07), impaired FBG (OR: 1.99, 95% CI: 1.33–3.01) and low HDL-C (OR: 15.92, 95% CI: 12.53–20.22). These associations remained significant after adjustment for age, gender, living area, socioeconomic status, physical activity, screen time and BMI.

**Table 4:** Odds ratio and 95% confidence interval for cardiometabolic risk factors across tertile categories of visceral adiposity index: the CASPIAN-V

**Tertilecategories of visceral adiposity index**	**Model 1**		**Model 2**		**Model 3**	
	**OR**	**95% CI**	**OR**	**95% CI**	**OR**	**95% CI**
obesity						
Tertile1 (reference)						
Tertile2	0.89	(0.69-1.15)	0.92	(0.70-1.22)		
Tertile3	1.07	(0.84-1.37)	1.14	(0.87-1.48)		
*P* for trend	0.56		0.33			
Abdominal obesity						
Tertile1 (reference)						
Tertile2	1.27	(1.04-1.57)	1.35	(1.08-1.68)		
Tertile3	1.69	(1.39-2.07)	1.77	(1.43-2.20)		
*P* for trend	<0.001		<0.001			
Impaired fasting glucose						
Tertile1(reference)						
Tertile2	1.52	(0.99-2.33)	1.61	(1.01-2.55)	1.64	(1.03-2.60)
Tertile3	1.99	(1.33-3.01)	1.98	(1.27-3.09)	2.00	(1.28-3.13)
*P* for trend	0.001		0.003		0.002	
Low HDL-C						
Tertile1(reference)						
Tertile2	4.50	(3.52-5.76)	4.48	(3.43-5.86)	4.49	(3.43-5.86)
Tertile3	15.92	(12.53-20.22)	15.93	(12.28-20.67)	15.93	(12.27-20.66)
*P* for trend	<0.001		<0.001		<0.001	
**High blood pressure**						
Tertile1(reference)						
Tertile2	1.20	(0.93-1.55)	1.27	(0.95-1.69)	1.29	(0.97-1.72)
Tertile3	1.03	(0.78-1.34)	1.15	(0.86-1.53)	1.14	(0.85-1.53)
*P* for trend	0.84		0.36		0.38	
High LDL-C						
Tertile1(reference)						
Tertile2	1.26	(1.02-1.54)	1.33	(1.06-1.66)	1.33	(1.06-1.66)
Tertile3	1.19	(0.97-1.46)	1.25	(1.01-1.57)	1.26	(1.01-1.57)
*P* for trend	0.11		0.053		0.052	

Model 1: Logistic regression crude model. Model 2: Adjustment for age, sex, living area, socioeconomic status, physical activity, screen time. Model 3: Additional adjustment for BMI.

The ROC curve for VAI for identifying children with MetS according to ATP III criteria is displayed in Table 5, stratified by gender and age groups. The cut-off points of the VAI were 1.58 in total participants (sensitivity: 79% and specificity: 78%), 1.30 (sensitivity: 76% and specificity: 81%) in boys, and 1.78 (sensitivity: 94% and specificity: 74%) in girls. In the 7-12 and 13-18 years age groups, these values were 1.59 (sensitivity: 76% and specificity: 81%) and 1.58 (sensitivity: 83% and specificity: 77%) in total population, 1.34 (sensitivity: 65% and specificity: 85%) and 1.58 (sensitivity: 76% and specificity: 87%) in boys, and 1.93 (sensitivity: 91% and specificity: 81%) and 1.58 (sensitivity: 76% and specificity: 87%) in girls, respectively.

**Table 5:** Receiver operator curve for visceral adiposity index for identifying children with MetS: the CASPIAN-V study

		**VAI cut-off points (95% CI)**	**Sensitivity (95% CI)**	**Specificity (95% CI)**	**AUC (95% CI)**
Boy	7-18 year	1.30 (1.05-1.55)	76% (66%-86%)	81% (72%-90%)	85% (81%-89%)
	7-12 year	1.34 (0.94-1.75)	65% (47%-83%)	85% (64%-100%)	82% (75%-88%)
	13-18 year	1.58 (1.24-1.93)	76% (63%-89%)	87% (75%-99%)	88% (83%-92%)
Girl	7-18 year	1.78 (1.41-2.14)	94% (86%-100%)	74% (65%-83%)	89% (87%-92%)
	7-12 year	1.93 (1.57-2.29)	91% (83%-99%)	81% (73%-90%)	91% (88%-94%)
	13-18 year	1.58 (1.24-1.93)	76% (63%-89%)	87% (75%-99%)	88% (84%-92%)
Total	7-18 year	1.58 (1.52-1.65)	79% (74%-86%)	78% (75%-82%)	84% (81%-87%)
	7-12 year	1.59 (1.19-1.99)	76% (64%-87%)	81% (72%-89%)	84% (79%-88%)
	13-18 year	1.58 (1.45-1.72)	83% (76%-91%)	77% (71%-83%)	84% (81%-88%)

CI: confidence interval; AUC: area under curve shown as percentage. Metabolic syndrome: ATP-III criteria; Abdominal obesity: WC> 90th percentile; Low HDL: HDL< 40 mg/dL (except in boys 15–19 y old, that cut-off was < 45 mg/dL); High TG: TG>100 mg/dL; High FBG: FBG > 100 mg/dL; High blood pressure: BP> 90th percentile (adjusted by age, sex, height).

**Figure. 1:**
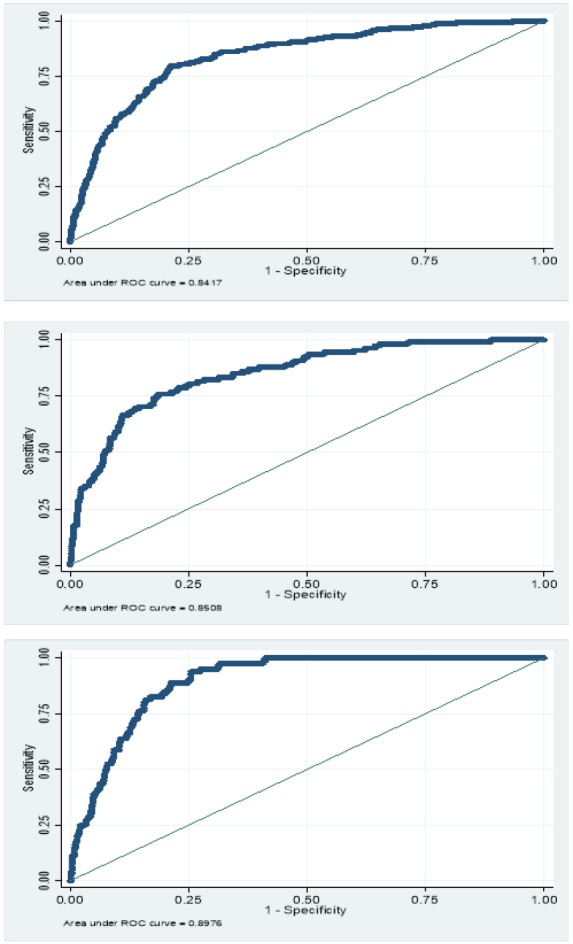
ROC curves for metabolic syndrome by sex. (A) ROC curves for metabolic syndrome in 7-18 years. (B) ROC curves for metabolic syndrome in boys. (C) ROC curves for metabolic syndrome in girls.
ROC, receiver operating characteristic.

The area under the curve (AUC) was 84% for total (in both age groups), 85% for boys (82% in 7-12 years; 88% in 13-18 years) and 89% for girls (91% in 7-12 years; 88% in 13-18 years) (Figure 1).

## Discussion

This is the first study to determine age- and gender-stratified cut-off points of VAI in Iranian children and adolescents, which proved to be strongly associated with MetS. The cut points of VAI for detecting MetS were 1.30 in boys, 1.78 in girls, 1.59 in 7-12 and 1.58 in 13-18 years age groups. We indicated that higher VAI scores is associated with 69% higher risk of abdominal obesity, 99% higher risk of impaired FBG and 15.92 times higher risk of low HDL-C in children and adolescents.

Regarding to increased prevalence of MetS among young population and its complications in adulthood, presenting more effective methods for early recognition of at risk groups is valuable.^21^ Abdominal obesity has been proved as a key component in the definition of MetS and WC is an important clinical factor used for indirect assessment of increased body fat and metabolic abnormalities in children and adolescents.^22-24^ However, distinguishing between subcutaneous and visceral fat mass is not possible by WC alone.^25^ Evidences suggest that different types of adipose tissue may exposed pediatrics to different risk factors of metabolic dysfunction. It has been demonstrated that increased visceral fat, plays a decisive role in appearance of metabolic abnormalities and cardiovascular disorders.^4,6,26^ In children, visceral fat has been shown to have a negative association with insulin sensitivity and HDL-C while positively associated with LDL-C, TG and insulin secretion.^27,28^ Taksali et al showed that risk of having MetS was significantly higher in adolescents with high visceral fat and low abdominal subcutaneous fat who was not necessarily severely obese.^26^ Since visceral fat measurement by using magnetic resonance imaging or euglycemic–hyperinsulinemic clamp are difficult to be performed in population-based survey, recent studies suggest VAI as a surrogate marker of indirect evaluation of visceral fat.^8,19^ VAI has been obtained and validated in adult populations and it has been shown that this index is independently associated with all MetS components, glucose values and cardio- and cerebrovascular events.^8-11,29^ Although these results are somehow in line with our finding, but studies regarding the calculation of this index and its association with metabolic abnormalities in children and adolescents are limited. To the best of our knowledge, only one study conducted in young population by Al- Daghri et al who obtained VAI of 543 children aged 4-17 years and showed that this index was significantly associated with BMI, WC, glucose, insulin resistance and SBP.^30^ We evaluated the applicability of VAI for identifying children with MetS and we observed that it could predict the risk of MetS in this age group too. However, more studies with a big cohort of children and adolescents would be required for modification and validation of VAI in this age group as a better clinical index for visceral adiposity and its related cardiometabolic disorders. Indeed, VAI has modified WC by combining some variables including BMI which indicates global adiposity and serum TG and HDL-C concentrations which represent of visceral adipose tissue function. Therefore, it seems that VAI present a better insight about function of visceral adipose tissue and insulin sensitivity than WC, and higher VAI score would be strongly correlated with MetS risk and cardiovascular complications. On the other hand, VAI declare other cardiometabolic risk factors which are not indicated by BMI, WC, TG, and HDL-C separately.^19^

### Study limitations

In the current study, we presented a gender and age-stratified cut-off of VAI in association with MetS for the first time in a large population of Iranian children and adolescents. However, there were some limitations. First, in terms of the relationship with MetS risks, in our survey we did not compare VAI with other direct or indirect measurements of visceral fat. Second, because of the cross-sectional nature of the present study, no causality can be concluded between VAI and MetS. Third, although pubertal stage affects insulin resistance and metabolic condition in children and adolescents, we have no information about the pubertal status of the participants.

## Conclusion

We determined the cut-off points of VAI for detecting MetS in Iranian children and adolescents. It was demonstrated that VAI is associated with cardiometabolic risk factors including abdominal obesity, impaired fasting glucose, low HDL-C, and high LDL-C in this age group of population. Therefore, VAI can be used as a surrogate marker of visceral adiposity and good indicator of MetS in pediatrics. However, prospective longitudinal studies are needed to show the possible efficiency of the VAI as a predictor of MetS and other cardiometabolic risk factors in children and adolescents.

## Competing interests

The authors declare that they have no competing interests.

## Ethical approval

After explanation, written and verbal consents were obtained from all the parents and students, respectively. The study was approved by the Research and Ethics Council of Isfahan University of Medical Sciences with ethical code of 194049.

## Acknowledgments

The authors are thankful of all participants and large team working on this project in different provinces. We appreciate Isfahan University of Medical Sciences and other relevant national regulatory organizations (Project number: 194049) for supporting this project.
